# In vivo assessment of cerebrospinal fluid efflux to nasal mucosa in humans

**DOI:** 10.1038/s41598-020-72031-5

**Published:** 2020-09-11

**Authors:** Erik Melin, Per Kristian Eide, Geir Ringstad

**Affiliations:** 1 Department of Radiology, Østfold Hospital Trust, Grålum, Norway; 2 Institute of Clinical Medicine, Faculty of Medicine, University of Oslo, Oslo, Norway; 3 Department of Neurosurgery, Oslo University Hospital-Rikshospitalet, Oslo, Norway; 4 Department of Radiology and Nuclear Medicine, Oslo University Hospital-Rikshospitalet, Oslo, Norway

**Keywords:** Neuroimmunology, Neuroscience, Neurology

## Abstract

Extra-vascular molecular clearance routes from the brain and cerebrospinal fluid (CSF) remain insufficiently characterized in humans. Animal studies consistently suggest that the cribriform plate and nasal lymphatic vessels are crucial for molecular clearance from CSF. In this study, we aimed to examine human in vivo transport of a CSF tracer from CSF to nasal mucosa. We hypothesised a CSF tracer would enrich in nasal mucosa provided that nasal lymphatic drainage has a significant role in CSF molecular clearance. Consecutive magnetic resonance imaging during 48 h after intrathecal administration of a tracer (gadobutrol) was performed in 24 patients. Despite a strong enrichment of CSF tracer in CSF spaces nearby the cribriform plate, there was no significant enrichment of CSF tracer in nasal mucosa, as measured in superior, medial and inferior turbinates, or in the nasal septum. Therefore, this in vivo study questions the importance of CSF drainage to the human nasal mucosa and emphasizes the need of further human studies.

## Introduction

There is limited knowledge about molecular efflux routes from cerebrospinal fluid (CSF) to lymphatic pathways in humans. Based on studies of a range of animal species, drainage to nasal mucosa via the cribriform plate is consistently shown to represent a major drainage route^1–10^. Hence, this route may be hypothesised to be of equal importance in humans. However, only one in vivo human study, utilizing radionuclide tracers, has been able to demonstrate possible clearance of molecules to the nasal turbinates, presumably via the cribriform plate^11^.

The recent reports of lymphatic vessels lining the dural sinuses^12,13^, and the possible connection between meningeal lymphatics and the glymphatic system^14^, have sparked a renewed interest in lymphatic drainage routes from CSF spaces. Experimentally induced impairment of meningeal lymphatic vessels suggests that lymphatic drainage is crucial for brain molecular clearance^13,15^, indicating a role of lymphatic drainage failure in neurodegeneration caused by the accumulation of neurotoxic molecules in brain tissue. Recently, our group used magnetic resonance imaging (MRI) to demonstrate the accumulation of a MRI contrast agent administered intrathecally in the human parasagittal dura, a tissue possibly serving as an intermediate step towards meningeal lymphatics, paralleled by efflux through neuroforamina at the skull base^16^. The methodology has yet not been used to assess whether this CSF tracer is cleared to nasal lymphatic efflux pathways.

In this study, we have therefore examined the efflux of intrathecal gadobutrol to nasal mucosa utilizing multi-phase, long-term MRI. We hypothesised that the MRI contrast agent gadobutrol, when administered intrathecally, enriches in nasal mucosa.

## Results

### Patients

The study included 24 patients examined with consecutive MRI scans before and after intrathecal administration of gadobutrol for work-up of tentative CSF circulation disorders. Patient data are given in Table 1.

**Table 1:** Patient material.

	Total material
N	24
Age (years)	41 ± 15
Gender (female/male)	18/6
BMI (kg/m^2^)	28 ± 5
**Clinical indication for MRI**	
iNPH	1 (4%)
Tentative spontaneous intracranial hypotension	3 (13%)
Arachnoid cyst	7 (29%)
Pineal cyst	6 (25%)
Idiopathic intracranial hypertension	7 (29%)

Data are given as numbers (percentage in parenthesis) and mean ± standard deviation.

### CSF tracer enrichment in CSF spaces

**Figure 1:**
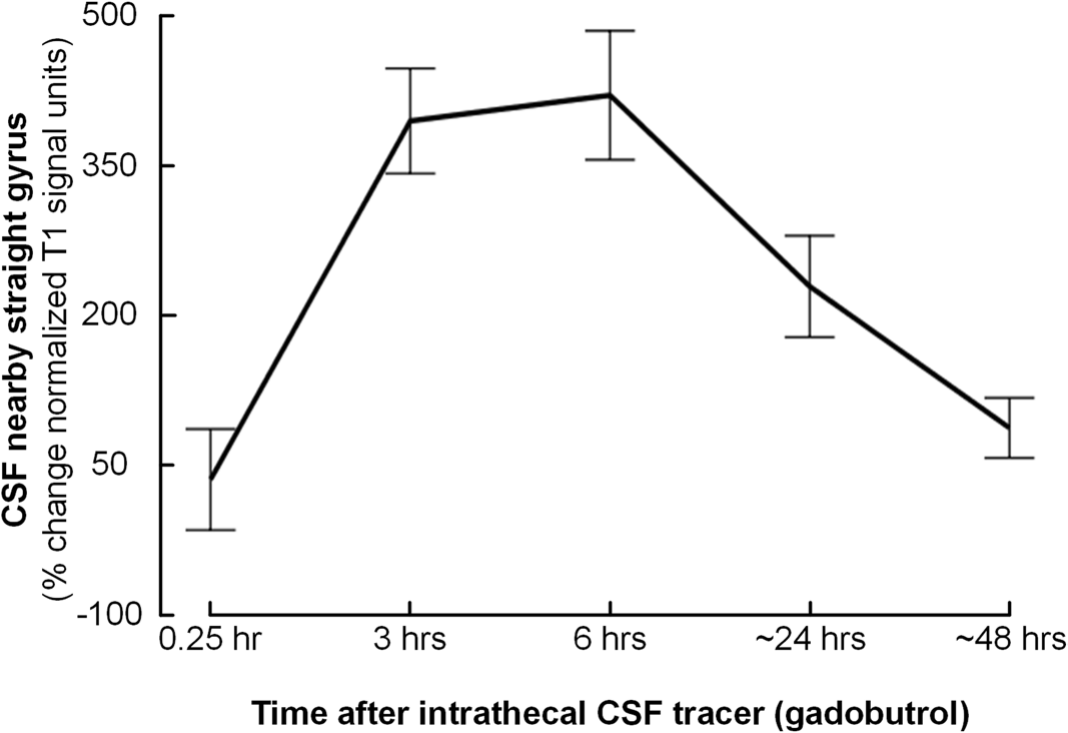
CSF tracer enrichment in CSF spaces. The percentage change in signal unit ratios over time is shown for CSF nearby the straight gyrus. The plot presents average percentage changes from the pre-tracer images with 95% confidence interval for every time point. Significance levels for the different regions of interest measured are presented in Table 2. There was a strongly significant enrichment of CSF tracer in this CSF space.

The tracer was detected in CSF close to the cribriform plate in all patients. There was a highly significant (P < 0.001) enrichment of CSF tracer in the subarachnoid space (SAS) adjacent to the straight gyrus with peak enrichment after 3–6 h (Fig. 1; Supplementary Table S1).

**Table 2:** Significance for percentage change in signal unit ratios for the different regions of interest.

Region of interest	Significance
Superior turbinates	0.252
Middle turbinates	0.302
Inferior turbinates	0.135
Nasal septum	0.676
Straight gyrus	< 0.001
White matter in frontal lobes	< 0.001
SAS adjacent to straight gyri	< 0.001
**Left side**	
Superior turbinate	0.045
Middle turbinate	0.322
Inferior turbinate	0.172
Nasal septum	0.68
Straight gyrus	< 0.001
White matter in frontal lobe	0.001
SAS adjacent to straight gyrus	< 0.001
**Right side**	
Superior turbinate	0.725
Middle turbinate	0.39
Inferior turbinate	0.1
Nasal septum	0.526
Straight gyrus	< 0.001
White matter in frontal lobe	< 0.001
SAS adjacent to straight gyrus	< 0.001

*SAS* subarachnoid space. Significance level was determined by mixed model analysis.

### CSF tracer enrichment inferior to the cribriform plate

**Figure 2:**
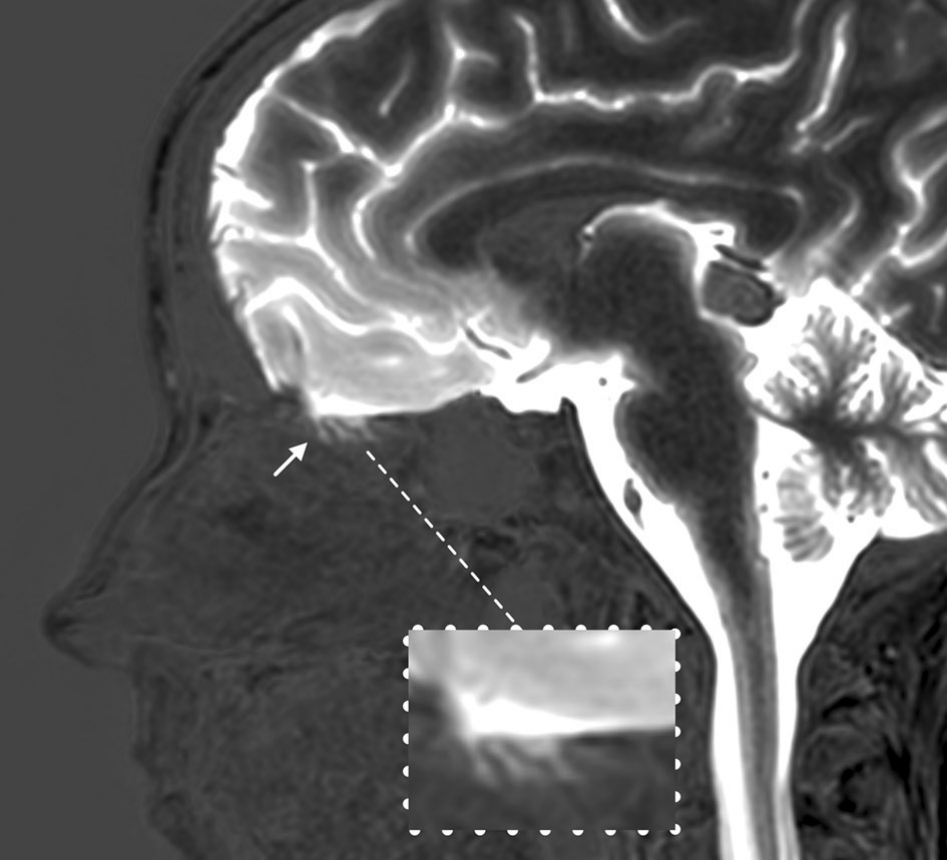
Subtraction magnetic resonance image visualizing tracer immediately below the cribriform plate. The illustration constitutes of a combination of T1-GRE images obtained at different time points: a summation of pre-contrast and 48 h’ post-contrast time points (with no or small amounts of CSF tracer) were subtracted from the 3- and 6-h’ time points (with high concentrations of tracer in CSF) to visualize the distribution of tracer. In this example, narrow streaks of tracer are depicted immediately below the cribriform plate (arrow). No tracer enhancement in the nasal mucosa can be readily seen and was confirmed by the ROI-analysis through all time points at group level.

Tracer was detected as minute tubular structures inferior to the cribriform plate 3–6 h after injection in 11 of 24 patients. Figure 2 is a post-processed image illustrating the distribution of tracer in one of the patients.

### CSF tracer enrichment in nasal mucosa

**Figure 3:**
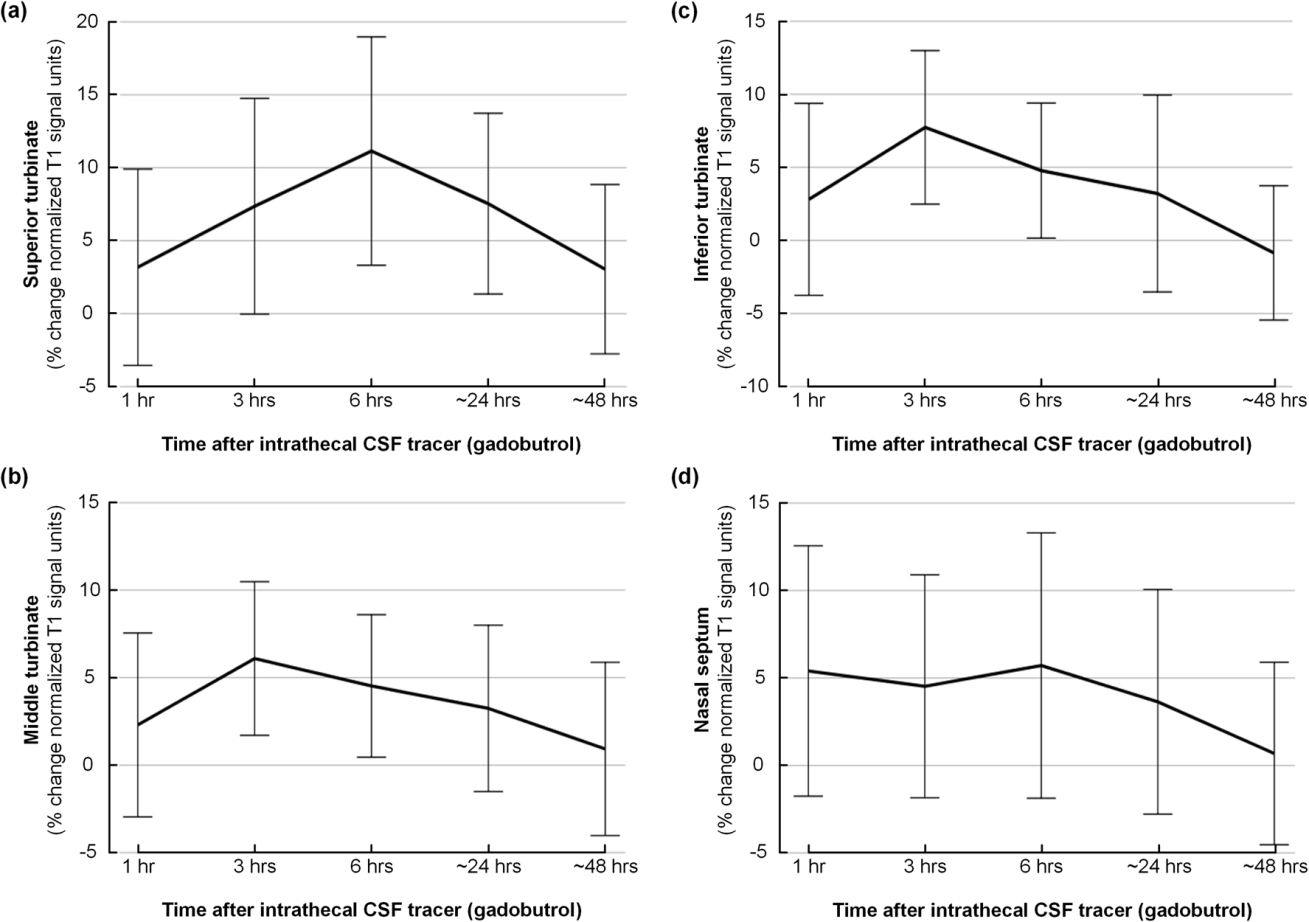
CSF tracer enrichment in nasal mucosa. The percentage change in signal unit ratios over time is shown for (**a**) superior turbinate, (**b**) middle turbinate, (**c**) inferior turbinate, and (**d**) nasal septum. The plots present average percentage changes from the pre-tracer images with 95% confidence interval for every time point. Significance levels for the different regions of interest measured are shown in Table 2. No significant enrichment of CSF tracer was observed in these regions.

The CSF tracer enrichment in different regions of interest within nasal mucosa at group level is presented in Fig. 3. The percentage change in signal unit ratio was not significant at any time point in any of the nasal cavity structures: (Fig. 3a) superior turbinate, (Fig. 3b) middle turbinate, (Fig. 3c) inferior turbinate and (Fig. 3d) nasal septum (see Supplementary Table S2–S5). Even though the signal unit ratio tended to increase 3 h after tracer administration for the middle and the inferior turbinates and after 6 h for the superior turbinate, the variation was large and not statistically significant.

### CSF tracer enrichment in brain parenchyma

**Figure 4:**
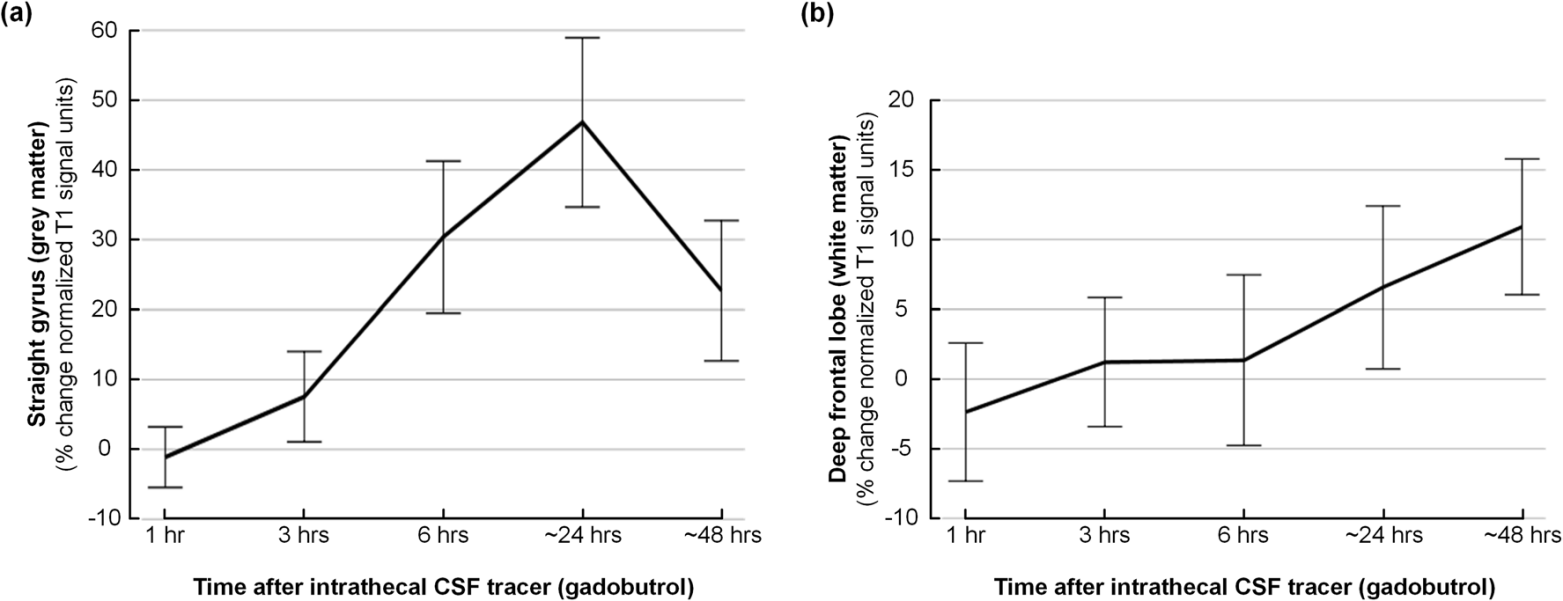
CSF tracer enrichment in brain parenchyma. The percentage change in signal unit ratios over time is shown for (**a**) straight gyrus and (**b**) deep white matter of the frontal lobe. The plots present average percentage changes from the pre-tracer images with 95% confidence interval for every time point. P-values for the different regions of interest measured are presented in Table 2, revealing significant CSF tracer enrichment within the brain parenchyma.

There was a highly significant (P < 0.001) enrichment of CSF tracer within the grey matter of the straight gyrus (Fig. 4a; Supplementary Table S6), as well as in the deep white matter of the frontal lobe (Fig. 4b; Supplementary Table S7).

### Safety of intrathecal contrast-enhanced MRI

No anaphylactic reactions, other serious adverse reactions or complications were noticed during or after the exams.

## Discussion

The main result of this study was that no CSF tracer could be detected in human nasal mucosa in vivo, raising the question of the relative importance of CSF clearance to lymphatic vessels in the nasal mucosa.

Lumbar intrathecal administration of the MRI contrast agent gadobutrol, serving as CSF tracer, has previously been shown to facilitate robust enrichment in the intracranial SAS compartment^17^. The time from injection until CSF tracer was present in cisterna magna was 20 ± 23 min in humans^18^. Enrichment of tracer in CSF is a requirement for enhancement in nearby cerebral structures^17,19,20^ as well as in parasagittal dura^16^. The present observations confirmed entry of tracer into the adjacent straight gyrus and strong enrichment of tracer in the SAS close to the cribriform plate. Tracer was also detected as minute tubular structures inferior to the cribriform plate in 11/24 patients (Fig. 2). Enrichment within nasal mucosa could be expected if efflux via the cribriform plate had a major role in human subjects.

Impaired clearance of toxic metabolites from CNS is considered an important mechanism behind neurodegenerative disease^21–25^. Recent discoveries have highlighted the perivascular route for clearance of macromolecules from the brain, such as β-amyloid and tau^14,24,26^, and meningeal lymphatic vessels seem to play an important role in brain and CSF clearance^12,13,15,16,27,28^. The dural lymphatic drainage route may be particularly important for a particularly toxic amyloid subtype^29^. The arachnoid is commonly considered as a barrier between CSF and the dura mater^30^. How molecules from CSF reach meningeal lymph vessels is not fully described, but in a previous study we detected a CSF tracer in parasagittal dura challenging the concept of an impermeable arachnoid^16^.

The tracer used in this study, gadobutrol, is of low molecular weight (604 Da), distributes freely in the CSF due to its hydrophilic properties and does not cross the blood–brain–barrier. As so, this MRI-tracer should be expected to follow extra-vascular clearance routes for molecules lacking specific transporters^31^.The movement of the tracer intracranially was assessed by our group in other studies^16,17,19,32^ and the tracer was also detected in the cervical lymph nodes after intrathecal administration^33^. However, molecules of low weight (< 5,000 Da) may also be absorbed by capillaries in the nasal mucosa^5^ possibly affecting the detectable amount of tracer in the area. Detection of the tracer in both deep cerebral white matter and in the straight gyrus of the frontal lobes confirmed that the applied T1-BB sequence enables for detection of tracer in limited amounts with good sensitivity.

In this study, we detected the CSF tracer as minute tubular structures inferior to the cribriform plate in 11/24 patients. This could represent tracer along perineural spaces, in lymphatic vessels or in the interstitial space^4^, or even tracer in arachnoid villi^34^. Perineural spread of tracer through the cribriform plate to nasal mucosa has previously been described in human^10,34^. In Johnston et al.^10^ a tracer injected in the SAS did indeed reach the lymphatic vessels of the nasal mucosa, both in animals and in one human, but no connection to lymphatic vessels was detected by Lowhagen et al.^34^. These studies provide good anatomical descriptions of the area, but do not necessarily reflect physiological conditions as they are performed post-mortem.

Our study findings contradicts De Leon et al., who utilized dynamic positron emission tomography (PET) to measure CSF clearance in humans and found significant levels of CSF tracer in the superior turbinates^11^. The PET technique used is probably more sensitive compared to MRI, but the image resolution of 4,5 mm is inferior to MRI (1 mm). The superior turbinates in humans are small structures, and the ROI used for superior turbinates in the PET-study may potentially have included adjacent areas. The ROI was also placed 5.2 mm inferior to the frontal lobe to avoid partial-volume effects from the brain, nevertheless, partial volume effects from CSF may have affected the measurements. Our results do confirm tracer inferior to the cribriform plate in a substantial proportion of patients (11/24) (Fig. 2), however, drainage to the nasal mucosa itself was not detected. The evidence of drainage to the middle turbinates provided with PET may be due to a higher sensitivity for detection of tracer in limited amounts. As molecules of low weight may to some extent be absorbed by capillaries before entering lymphatic vessels in nasal mucosa^5^, we anticipate such “leakage” of tracer not to be very different in these contradictive studies, as De Leon et al. also used molecules with low weight (< 328 Da).

Both animal and human studies suggest transport of molecules in the opposite direction, that is, from nasal mucosa to CSF, following anatomical pathways along the olfactory and trigeminal nerves bypassing the blood–brain barrier ^35^. Substances have been detected in CSF within 30 min after intranasal administration^36^. One plausible theoretical explanation for such rapid transport is extracellular (perineural or paravascular) convective flow^37^.

From an anatomical point of view, the human bipedal position, and the small olfactory area, which may be considered to be of a rather rudimentary size compared to rodents^38,39^, could have an impact on species-dependent differences in trans-nasal CSF molecular efflux. We, therefore, hypothesise that CSF molecular efflux through other foramina in the skull base and meningeal lymphatics^16,27,30^ could be of more importance in humans compared to many other species. Other postulated molecular efflux routes are via the arachnoid villi to dural venous sinuses^40^ and across the blood–brain barrier^41^.

This study included patients with various CSF-disorders (Table 1). 13/24 patients were examined for intracranial cysts not adjacent to the cribriform plate. It is unlikely that these patients’ efflux systems in any way would be compromised or affected by the cysts. 8/24 were examined for idiopathic normal pressure hydrocephalus (iNPH) or idiopathic intracranial hypertension. As the pathophysiology of these latter disorders is not understood, the CSF dynamics in these patients could possibly be affected. 3/24 were examined for tentative spontaneous intracranial hypotension. No CSF leakage was seen in these patients, but as for iNPH and idiopathic intracranial hypertension, these patients present with pathology that potentially could have affected the results.

The temporal resolution of imaging was low, particularly for late scans. However, MRI scanning was continued throughout a considerable time frame and beyond the time of peak tracer enhancement in CSF. It seems therefore unlikely that mucosal enrichment followed by an expectedly slow molecular escape should have occurred entirely in intervals between MRI scans.

The connections between the SAS, perineural spaces, lymphatic vessels and interstitial spaces in the nasal mucosa are not clarified^4^. Visualization of individual lymphatic vessels or perineural spaces in the nasal mucosa using MRI cannot be expected due to the MR image resolution (1 × 1 × 1 mm); the circular ROIs were positioned to measure from representative samples of the mucosa/submucosa covering perineural spaces, lymphatic vessels and the interstitial space. The concentration of the tracer in nasal mucosa would be expected to be lower than in the CSF after dilution from lymph and interstitial fluid in the nasal mucosa. Artefacts from both movement and surrounding areas may have decreased the signal-to-noise ratio and confounded the measurements even if obvious artefacts were avoided and images disturbed by motion were excluded. To this end, our negative result could be explained by low concentrations of tracer undetectable by the methodology utilized in this study.

**Figure 5:**
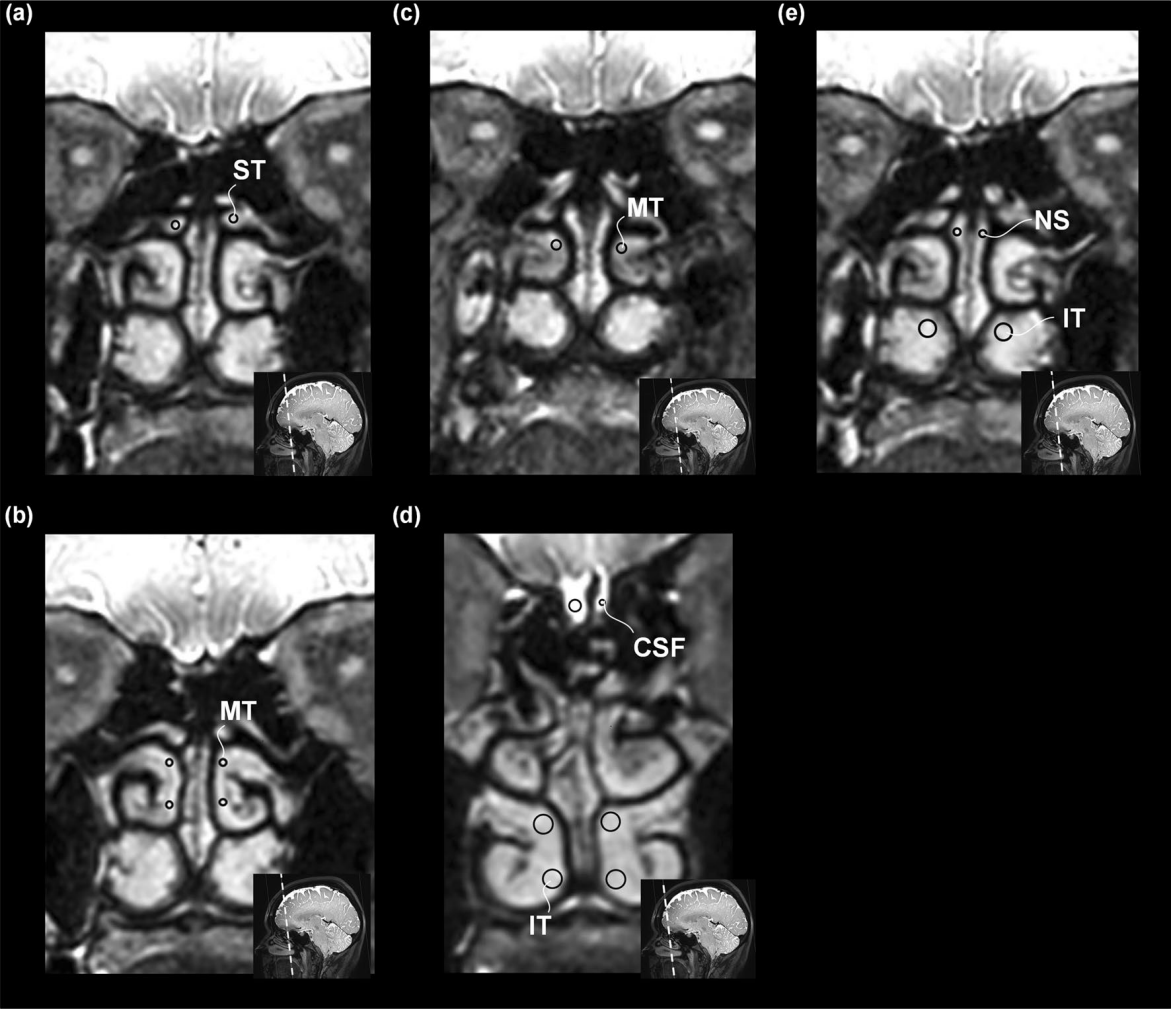
Slice sections and measurement areas on T1-weighted blood suppressed magnetic resonance images. Coronal images of nasal mucosa illustrate the various regions of interest (ROIs) assessed in this study; each ROI is indicated by a circle. The slice section is shown in the lower right corner of each image. Regions of interest indicated by a circle are shown for the following structures (**a**) superior turbinate (ST), (**b**) middle turbinate (MT), (**c**) middle turbinate (MT) here illustrated by the posterior part, (**d**) inferior turbinate (IT), also showing CSF nearby straight gyri, (**e**) nasal septum (NS) and posterior part of inferior turbinate (IT).

For some measurements, the signal intensity in the CSF, nasal mucosa and brain parenchyma seemingly declined after contrast agent administration compared to pre-contrast images (Figs. 3, 4 and 5). Since this is implausible, the most likely explanation for this is artefacts affecting the signal within any of the ROIs, including the reference ROIs and the ROIs in the CSF, nasal mucosa and brain parenchyma.

Diurnal volume fluctuations of the nasal mucosa derive from dilation and constriction of the venous cavernous tissue in the turbinates^42^. Therefore, ROI locations had to be manually adapted to local volume changes to avoid misregistration and partial volume effects from adjacent regions, particularly from the airways. The volume differences represent to some extent differences in blood volume. The intrinsic signal in nasal mucosa could thus potentially change for other reasons than the enrichment of tracer. The volume differences in the nasal mucosa also limited the possible regions to include in the measurements. On the other hand, volume changes would be random, and should not produce a systematic effect of the changes in MRI signal units in one direction. Even though manual placement of ROIs may not be optimal, and potentially biased, this was considered the only feasible option due to the inter- and intrapatient physiological fluctuations.

In conclusion, this study did not confirm clearance of CSF tracer to the nasal mucosa in humans, unlike in many previous animal studies, thus questioning the importance of this molecular clearance route in humans. The method applied in this study has limitations, particularly concerning the use of a small molecular weight CSF tracer. Future studies are needed to address to which extent molecular efflux to nasal mucosa differ between animals and humans.

## Methods

### Subjects and study design

The study design was prospective and observational. Patients referred to the Department of neurosurgery, Oslo University Hospital-Rikshospitalet were included for consecutive MRI exams after intrathecal administration of the contrast agent gadobutrol. Inclusion criteria were tentative CSF circulation disorders. Exclusion criteria were age < 18 years or > 80 years, pregnancy and breastfeeding, known adverse reaction to contrast media, history of severe allergic reaction in general, and renal dysfunction. A total number of 34 patients were included for imaging. After imaging, 10 subjects were excluded due to movement artefacts, missing MRI exams, or signs of rhinitis at MRI. Of the remaining 24 patients, 12 were included in a previous study assessing tracer enrichment in the parasagittal dura^16^.

MRI was obtained approximately 8 a.m. followed by an X-ray guided lumbar puncture performed by an experienced interventional neuroradiologist. Backflow from the puncture needle verified a correct position in the SAS. A radiopaque contrast agent, typically 3 ml of 270 mg I/ml iodixanol (Visipaque, GE Healthcare), was injected to confirm unrestricted passage in the lumbar SAS. Thereafter, 0.5 ml of 1.0 mmol/ml gadobutrol (Gadovist, Bayer AB, Sweden) was administered. After needle removal, the study subjects were instructed to rotate themselves around the long axis of the body once and remain in a supine position for transportation back to the MRI suite for the first post-contrast image.

### MRI protocol

A 3 T Philips Ingenia MRI scanner (Philips Medical Systems) with a 32-channel head coil was used with equal imaging protocol settings at all time points to acquire whole-brain sagittal T1-weighted volume scans with suppression of intraluminal blood signal (“Black Blood”) (T1-BB). The parameters were TR/TE = 700/35 ms, echo train length = 55, flip angle = 80°, 2 averages, 1 × 1 × 1 mm voxel size (isotropic). Acquisition time was 4 min and 54 s.

For practical reasons, the consecutive MRI exams could not be obtained at identical time points in every subject. The MRI exams were therefore categorized into the following approximate time points according to an intention-to-scan basis: pre-contrast, < 1 h (mean 18 min, SD ± 5 min) after contrast, 3 h (mean 3 h and 7 min, SD ± 20 min) after contrast, 6 h (mean 6 h and 2 min, SD ± 24 min) after contrast, 24 h (mean 25 h and 2 min, SD ± 1 h and 13 min) after contrast and 48 h (mean 47 h and 58 min, SD ± 1 h and 25 min) after contrast. The patients were instructed to stay in bed until the 6 h’ time point and were allowed to move freely thereafter.

Whole-brain 3D T1-gradient echo (GRE) images were also obtained during each imaging session. The imaging parameters were as follows: TR = shortest (typically 5.1 ms), echo time = shortest (typically 2.3 ms), echo train length = 232, flip angle = 8°, 1 average, 1 × 1 × 1 mm voxel size and acquisition time 6 min and 29 s.

T1-BB scans were deemed superior to T1-GRE with regards to having a better signal-to-noise ratio in the region of the nasal mucosa and were chosen for the ROI-analysis in all areas of interest. The T1-GRE images were used for visually detection of tracer inferior to the cribriform plate and in elaboration of the image showing the tracer immediately below the cribriform plate (Fig. 2).

### Intrathecal administration of gadolinium

Gadobutrol is approved for intravenous use only by the United States’ Food and Drug Administration (FDA). Intrathecal administration of gadobutrol has so far been used off-label in a controlled study setting at our institution in patients with specific clinical indications for work-up of CSF circulation disorders. Gadobutrol is a macrocyclic gadolinium contrast agent. Concerns about gadolinium brain deposition have primarily been associated with linear contrast agents^43^. Recently published data showed the same safety profile for intrathecal administration of gadobutrol as for intrathecal administration of the CT contrast agent iodixanol^18^, and no signs of deposition in brain tissue have been detected after 4 weeks^19^.

### Image analysis

All T1-BB volume scans were reconstructed to 1 mm thick coronal sections perpendicular to a line between the anterior and posterior commissure. A board-certified neuroradiologist (EM) with 7 years’ experience in neuroradiology manually placed 24 circular, pre-defined regions of interest (ROIs) at all time points using the hospital’s Picture Archiving and Communication System (SECTRA IDS7, Sweden). The placements of the ROIs in the nasal mucosa and the SAS in one patient are illustrated in Fig. 5. The placements of the ROIs in the brain parenchyma can be found in Supplementary Fig. S2.

The ROIs in the nasal turbinates, including the posterior parts, were based on anatomical considerations in rodents^2^, a study of lymphatic vessels in humans^44^, post-mortem studies of humans^10^ and the PET-study by de Leon et al.^11^ to be as anatomically correct as possible for detection of tracer in lymphatic vessels. The lymphatic vessels were expected to be of size far below the image resolution of 1 mm, and therefore not directly detectable.

The exact placement and size of each set of ROIs were chosen with the intention to avoid artefacts and to cover as much relevant tissue as possible and at the same time striving to be anatomically stringent between different time points. 16 of the ROIs were placed in the mucosa/submucosa of the nasal turbinates and nasal septum as follows: 3 ROIs were placed bilaterally on each middle and inferior turbinate including the posterior parts of the turbinates; 1 ROI bilaterally on the superior turbinates; 1 ROI on both sides in the superior part of the nasal septum. 8 ROIs outside of the nasal cavity was measured: bilaterally in the deep white matter of the frontal lobes; bilaterally in straight gyrus of the frontal lobe and the adjacent SAS, respectively.

In addition, ROIs were placed bilaterally in the vitreous body of the ocular bulb for reference (Supplementary Fig. S1). The average pixel intensity for each ROI was measured. Further, we divided the average pixel intensity against the average pixel intensity in the reference ROIs placed within the vitreous body bilaterally in the coronal reconstructed images from the same T1-BB scan. Any significant tracer accumulation in the vitreous body after intrathecal administration was considered highly unlikely. We refer to this method as normalization of pixel intensity; the process corrects for any baseline changes of image greyscale due to image scaling.

Another board-certified neuroradiologist (GR) with 13 years’ experience in neuroradiology verified the placement of the ROIs and the overall quality of the images.

The 3D T1-GRE images were visually analysed in reconstructed 1 mm thick coronal and sagittal images for tracer inferior to the cribriform plate in all 24 patients for all time points. The 3D T1-GRE images were also used as source data for Fig. 2: the pre-contrast images were segmented using SPM12 (The Wellcome Centre for Human Neuroimaging, London, UK) and images from different time points were aligned in 3D slicer (version 4, www.slicer.org)^45^. Figure 2 is composed of a combination of images from one patient: pre-contrast and the 48 h’ time points were subtracted from the 3 and 6 h’ timepoints to enhance differences between the images; In this case to visualize the distribution of the tracer 3 and 6 h after contrast.

### Statistics

SPSS software version 24 (IBM Corporation, Armonk, NY, USA) was used for statistical analyses. Linear mixed model analysis was used to assess whether changes in signal unit ratios over time were statistically significant. Pearson Chi-square test was used to determine differences between categorical data. Statistical significance at the 0.05 level (two-tailed) was accepted.

### Ethical approvals

The study was approved by the Institutional Board of Oslo University Hospital (2015/1868), the Regional Ethical Committee of South-East Norway (2015/96) and the National Medicines Agency (15/04932-7). The protocol was conducted in accordance with the relevant guidelines and regulations. Oral and written informed consent was obtained from all participants.

### Supplementary information

Supplementary file1
