# Author Correction: In vivo assessment of cerebrospinal fluid efflux to nasal mucosa in humans

**DOI:** 10.1038/s41598-023-43372-8

**Published:** 2023-10-03

**Authors:** Erik Melin, Per Kristian Eide, Geir Ringstad

**Affiliations:** 1 Department of Radiology, Østfold Hospital Trust, Grålum, Norway; 2 Institute of Clinical Medicine, Faculty of Medicine, University of Oslo, Oslo, Norway; 3 Department of Neurosurgery, Oslo University Hospital-Rikshospitalet, Oslo, Norway; 4 Department of Radiology and Nuclear Medicine, Oslo University Hospital-Rikshospitalet, Oslo, Norway

Correction to: *Scientific Reports* https://doi.org/10.1038/s41598-020-72031-5, published online 11 September 2020

The original version of this Article omitted an affiliation for Erik Melin. The correct affiliations are listed below.

Department of Radiology, Østfold Hospital Trust, Grålum, Norway

Institute of Clinical Medicine, Faculty of Medicine, University of Oslo, Oslo, Norway

The original Article has been corrected.

