# Supplementary material for: In vivo assessment of cerebrospinal fluid efflux to nasal mucosa in humans
Source: Sci Rep. 2020 Sep 11;10:14974. doi: 10.1038/s41598-020-72031-5 (PMC7486888; doi:10.1038/s41598-020-72031-5)
Supplement: Supplementary file 1 — Supplementary file1 [file 41598_2020_72031_MOESM1_ESM.pdf]

Supplementary Information

In vivo assessment of cerebrospinal fluid efflux to nasal mucosa in humans

Erik Melin[a], Per Kristian Eide[b,c,*], Geir Ringstad[d]

[a]*Dept. of Radiology, Østfold Hospital Trust, Grålum, Norway,* [b]*Dept. of Neurosurgery, Oslo University Hospital-Rikshospitalet,* [c]*Institute of Clinical Medicine, Faculty of Medicine, University of Oslo, Oslo, Norway,* [d]*Dept. of Radiology and Nuclear Medicine, Oslo University Hospital - Rikshospitalet, Norway*

***Corresponding author:**

Email: p.k.eide@medisin.uio.no (PKE)

Supplementary Figure S1. Reference Regions of Interest

We used the vitreous body of the ocular bulb for reference. Circular regions of interest (ROIs) were manually drawn in the centres of the vitreous bodies from the same stack of coronal T1-BB MRI used for assessment of the nasal mucosa, CSF and brain parenchyma. The average signal intensity in each ROI in the nasal mucosa, CSF and brain parenchyma was divided by the reference ROIs to correct for any changes in grayscale differences between the time points. Artefacts were avoided when visually detected.

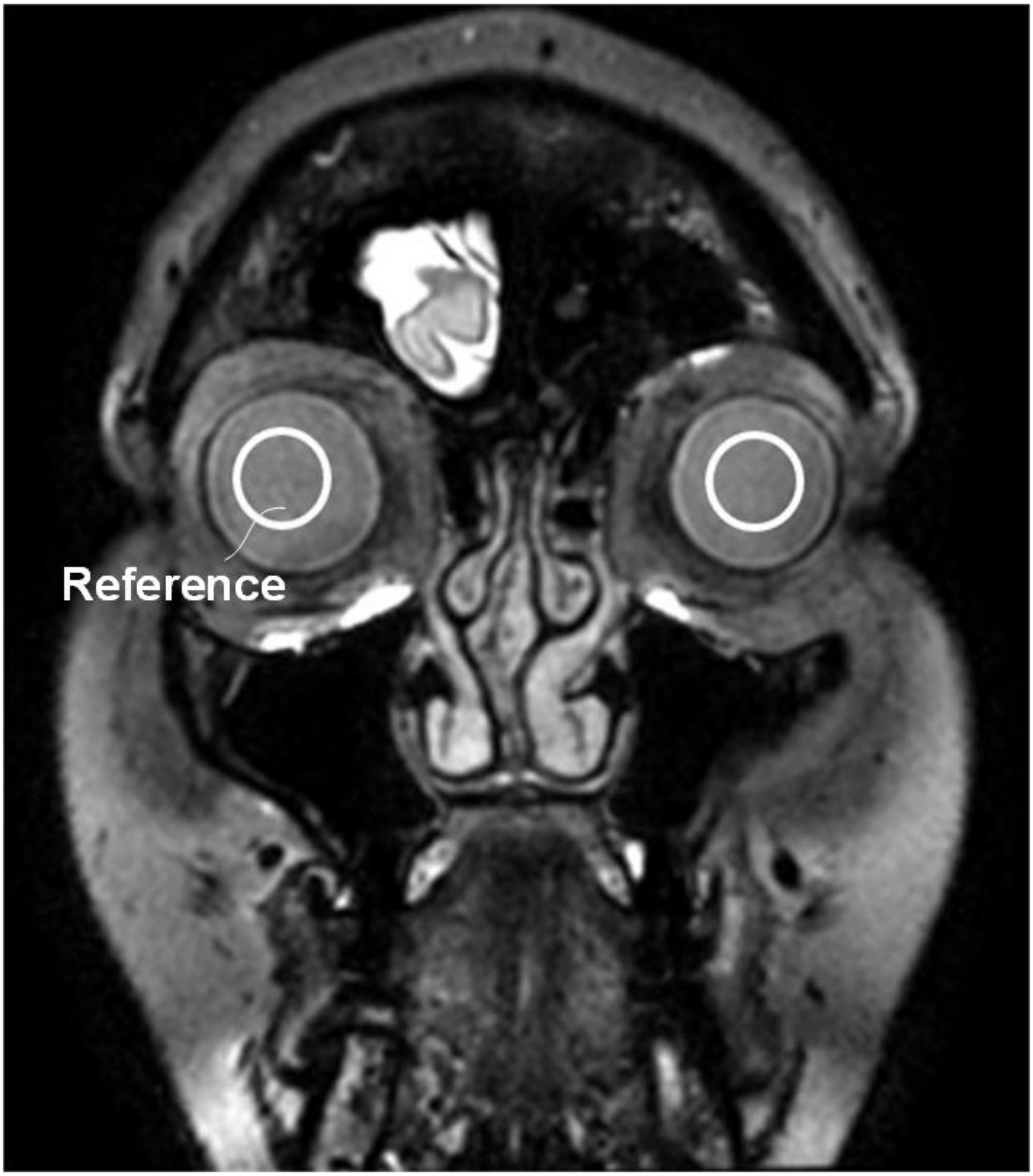

Supplementary Figure S2. Brain Parenchyma Regions of Interest

Circular regions of interest (ROIs) were manually placed bilaterally in (a) the straight gyri and (b) in the deep white matter of the frontal lobes. The same stack of coronal T1-BB MRI used for assessment of the nasal mucosa, CSF and brain parenchyma was used. Artefacts in the straight gyri from the nearby sphenoidal sinus were evident in a number of patients. These artefacts were avoided by placing the ROIs in the superior parts of the straight gyri. Slice sections are shown in the small sagittal images in the lower right corner of each image.

(a)

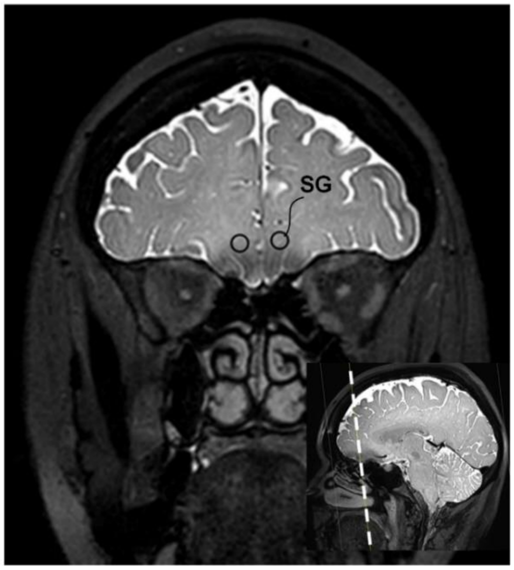

(b)

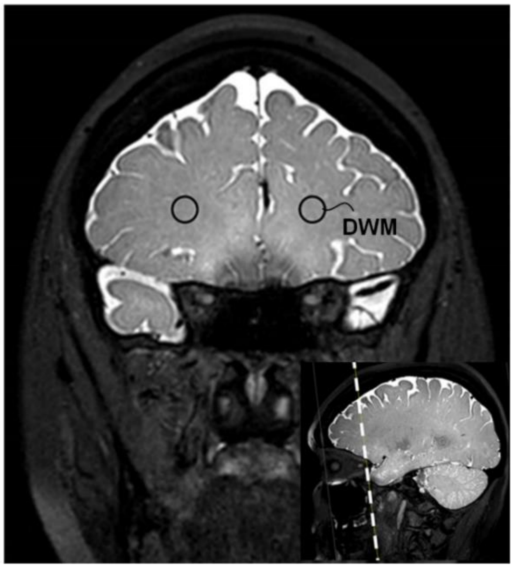

Supplementary Table 1. Signal intensity within cerebrospinal fluid and reference tissue including normalized signal unit ratios, before (Pre) and after intrathecal gadobutrol

	Pre contrast			Time after intrathecal gadobutrol															
				0.25 hours			3 hours			6 hours			24 hours			48 hours		
**Patient**	SU_{CSF}	SU_{REF}	R	SU_{CSF}	SU_{REF}	R	SU_{CSF}	SU_{REF}	R	SU_{CSF}	SU_{REF}	R	SU_{CSF}	SU_{REF}	R	SU_{CSF}	SU_{REF}	R
1	180	118	1.5	189	112	1.7	783	86	9.2	719	80	9.0	623	94	6.6	359	119	3.0
2	195	108	1.8				844	90	9.4	749	83	9.1	192	104	1.9	206	105	2.0
3	172	112	1.5				817	91	9.0	833	94	8.9	472	125	3.8	275	119	2.3
4	156	110	1.4				658	89	7.4	731	93	7.9	356	118	3.0	226	122	1.9
5	165	100	1.6				532	85	6.3	682	75	9.1	622	111	5.6	266	106	2.5
6	168	132	1.3				670	90	7.5	653	83	7.9	691	96	7.2	322	113	2.9
7	145	109	1.3	143	111	1.3	675	93	7.3	733	86	8.5	493	114	4.3	219	106	2.1
8	160	99	1.6		64		618	64	9.7	675	79	8.5	448	93	4.8	262	109	2.4
9	136	99	1.4	102	80	1.3	350	83	4.2				441	77	5.7	209	77	2.7
10	163	96	1.7	184	106	1.7	762	77	10.0				425	98	4.3	195	107	1.8
11	149	97	1.5	143	97	1.5	677	88	7.7	634	77	8.2	255	102	2.5	169	95	1.8
12	175	109	1.6	162	112	1.4	412	105	3.9	635	95	6.7	759	101	7.5	521	110	4.8
13	190	107	1.8	173	98	1.8	478	90	5.3	646	105	6.2	410	110	3.7		114	
14	152	100	1.5	144	107	1.4	597	83	7.2	746	83	9.0	715	94	7.6	469	115	4.1
15	158	114	1.4	564	97	5.8	775	89	8.8	810	83	9.8	463	103	4.5	335	105	3.2
16	129	89	1.4	150	105	1.4	706	78	9.1	797	88	9.1	280	112	2.5	235	104	2.3
17	188	108	1.7	535	110	4.9	747	93	8.0	825	101	8.2	594	114	5.2	261	107	2.4
18	102	76	1.3				385	80	4.8	578	100	5.8	469	95	5.0	213	76	2.8
19	145	103	1.4				688	95	7.3	750	88	8.6	663	91	7.3	535	96	5.6
20	168	93	1.8	161	92	1.7	524	87	6.0	690	93	7.5	654	96	6.8	454	100	4.5
21	145	105	1.4	192	99	1.9	806	88	9.2				592	109	5.5	287	110	2.6
22	178	98	1.8	176	91	1.9	586	68	8.6	709	71	10.0	685	86	8.0	384	93	4.2
23	141	110	1.3	121	95	1.3	797	99	8.1	828	97	8.5	424	120	3.5	225	129	1.7
24	151	93	1.6							844	88	9.6	315	93	3.4	171	96	1.8

**Mean ± STD**	**159 ± 21**	**103 ± 11**	**1.5 ± 0.2**	**209 ± 140**	**98 ± 13**	**2.1 ± 1.4**	**647 ± 144**	**86 ± 9**	**7.6 ± 1.8**	**727 ± 76**	**87 ± 9**	**8.4 ± 1.1**	**501 ± 157**	**102 ± 12**	**5.0 ± 1.8**	**295 ± 110**	**105 ± 13**	**2.8 ± 1.1**

SU = signal unit; CSF = CSF within Sylvian fissure close to frontal inferior gyrus; REF = vitreous body of the eye; R = signal units within CSF divided by signal units within REF; STD = standard deviation.

Supplementary Table 2. Signal intensity within superior turbinate and reference tissue including normalized signal unit ratios, before (Pre) and after intrathecal gadobutrol

	Pre contrast			Time after intrathecal gadobutrol														
				0.25 hours			3 hours			6 hours			24 hours			48 hours		
**Patient**	**SU_{ST}**	**SU_{REF}**	**R**	**SU_{ST}**	**SU_{REF}**	**R**	**SU_{ST}**	**SU_{REF}**	**R**	**SU_{ST}**	**SU_{REF}**	**R**	**SU_{ST}**	**SU_{REF}**	**R**	**SU_{ST}**	**SU_{REF}**	**R**
1	156	118	1.3	170	112	1.5	131	86	1.5	143	80	1.8	180	94	1.9	197	119	1.7
2	164	108	1.5				140	90	1.6	148	83	1.8	169	104	1.6	165	105	1.6
3	161	112	1.4				187	91	2.1	129	94	1.4	171	125	1.4	190	119	1.6
4	156	110	1.4				164	89	1.8	157	93	1.7	185	118	1.6	168	122	1.4
5	194	100	1.9				148	85	1.7	159	75	2.1	211	111	1.9	226	106	2.1
6	205	132	1.6				181	90	2.0	175	83	2.1	187	96	2.0	209	113	1.9
7	187	109	1.7	190	111	1.7	136	93	1.5	171	86	2.0	167	114	1.5	177	106	1.7
8	189	99	1.9		64		147	64	2.3	165	79	2.1	184	93	2.0	222	109	2.0
9	186	99	1.9	157	80	2.0	156	83	1.9				171	77	2.2	150	77	1.9
10	154	96	1.6	182	106	1.7	117	77	1.5				189	98	1.9	179	107	1.7
11	148	97	1.5	172	97	1.8	157	88	1.8	163	77	2.1	141	102	1.4	174	95	1.8
12	181	109	1.7	208	112	1.9	190	105	1.8	192	95	2.0	201	101	2.0	186	110	1.7
13	159	107	1.5	180	98	1.8	142	90	1.6	162	105	1.5	195	110	1.8		114	
14	161	100	1.6	183	107	1.7	124	83	1.5	104	83	1.3	173	94	1.8	166	115	1.4
15	217	114	1.9	156	97	1.6	159	89	1.8	185	83	2.2	180	103	1.8	169	105	1.6
16	127	89	1.4	151	105	1.4	118	78	1.5	121	88	1.4	146	112	1.3	178	104	1.7
17	160	108	1.5	136	110	1.2	127	93	1.4	126	101	1.2	190	114	1.7	155	107	1.4
18		76						80			100			95			76	
19	167	103	1.6				126	95	1.3	149	88	1.7	134	91	1.5	121	96	1.3
20		93			92			87			93			96			100	
21	137	105	1.3	119	99	1.2	122	88	1.4				154	109	1.4	141	110	1.3
22	140	98	1.4	126	91	1.4	117	68	1.7	118	71	1.7	137	86	1.6	148	93	1.6
23	225	110	2.0	201	95	2.1	245	99	2.5	217	97	2.2	248	120	2.1	218	129	1.7
24	144	93	1.6							144	88	1.6	155	93	1.7	152	96	1.6

**Mean ± STD**	**169 ± 26**	**103 ± 11**	**1.6 ± 0.2**	**166 ± 27**	**98 ± 13**	**1.6 ± 0.3**	**149 ± 31**	**86 ± 9**	**1.7 ± 0.3**	**154 ± 28**	**87 ± 9**	**1.8 ± 0.3**	**176 ± 26**	**102 ± 12**	**1.7 ± 0.3**	**176 ± 28**	**105 ± 13**	**1.7 ± 0.2**

SU = signal unit; ST = superior turbinate; REF = vitreous body of the eye; R = signal units within superior turbinate divided by signal units within REF; STD = standard deviation.

Supplementary Table 3. Signal intensity within middle turbinate and reference tissue including normalized signal unit ratios, before (Pre) and after intrathecal gadobutrol

	Pre contrast			Time after intrathecal gadobutrol														
				0.25 hours			3 hours			6 hours			24 hours			48 hours		
**Patient**	**SU_{MT}**	**SU_{REF}**	**R**	**SU_{MT}**	**SU_{REF}**	**R**	**SU_{MT}**	**SU_{REF}**	**R**	**SU_{MT}**	**SU_{REF}**	**R**	**SU_{MT}**	**SU_{REF}**	**R**	**SU_{MT}**	**SU_{REF}**	**R**
1	200	118	1.7	183	112	1.6	143	86	1.7	142	80	1.8	178	94	1.9	220	119	1.9
2	164	108	1.5				161	90	1.8	133	83	1.6	143	104	1.4	166	105	1.6
3	148	112	1.3				148	91	1.6	131	94	1.4	167	125	1.3	151	119	1.3
4	156	110	1.4				139	89	1.6	152	93	1.6	189	118	1.6	179	122	1.5
5	230	100	2.3				173	85	2.0	163	75	2.2	229	111	2.1	218	106	2.1
6	223	132	1.7				166	90	1.9	163	83	2.0	214	96	2.2	202	113	1.8
7	189	109	1.7	191	111	1.7	141	93	1.5	170	86	2.0	202	114	1.8	192	106	1.8
8	185	99	1.9		64		142	64	2.2	140	79	1.8	160	93	1.7	211	109	1.9
9	140	99	1.4	143	80	1.8	145	83	1.7	77			136	77	1.8	145	77	1.9
10	191	96	2.0	203	106	1.9	149	77	2.0				178	98	1.8	174	107	1.6
11	196	97	2.0	207	97	2.1	182	88	2.1	180	77	2.3	200	102	2.0	219	95	2.3
12	199	109	1.8	209	112	1.9	193	105	1.8	164	95	1.7	188	101	1.9	205	110	1.9
13	146	107	1.4	155	98	1.6	128	90	1.4	165	105	1.6	147	110	1.3		114	
14	184	100	1.8	204	107	1.9	158	83	1.9	146	83	1.8	197	94	2.1	210	115	1.8
15	223	114	2.0	175	97	1.8	190	89	2.1	186	83	2.3	190	103	1.9	191	105	1.8
16	172	89	1.9	202	105	1.9	165	78	2.1	155	88	1.8	192	112	1.7	200	104	1.9
17	145	108	1.3	133	110	1.2	134	93	1.4	142	101	1.4	184	114	1.6	176	107	1.6
18	123	76	1.6				133	80	1.7	159	100	1.6	162	95	1.7	116	76	1.5
19	176	103	1.7				146	95	1.5	139	88	1.6	158	91	1.7	165	96	1.7
20	167	93	1.8	159	92	1.7	161	87	1.8	173	93	1.9	171	96	1.8	168	100	1.7
21	181	105	1.7	162	99	1.6	151	88	1.7				179	109	1.7	161	110	1.5
22	176	98	1.8	172	91	1.9	144	68	2.1	143	71	2.0	171	86	2.0	170	93	1.8
23	204	110	1.9	194	95	2.0	210	99	2.1	195	97	2.0	225	120	1.9	214	129	1.7
24	168	93	1.8							153	88	1.7	167	93	1.8	164	96	1.7

**Mean ± STD**	**178 ± 28**	**103 ± 11**	**1.7 ± 0.2**	**179 ± 25**	**98 ± 13**	**1.8 ± 0.2**	**157 ± 21**	**86 ± 9**	**1.8 ± 0.2**	**153 ± 24**	**87 ± 9**	**1.8 ± 0.3**	**180 ± 24**	**102 ± 12**	**1.8 ± 0.2**	**183 ± 27**	**105 ± 13**	**1.8 ± 0.2**

SU = signal unit; MT = middle turbinate; REF = vitreous body of the eye; R = signal units within middle turbinate divided by signal units within REF; STD = standard deviation.

Supplementary Table 4. Signal intensity within inferior turbinate and reference tissue including normalized signal unit ratios, before (Pre) and after intrathecal gadobutrol

Pre contrast				Time after intrathecal gadobutrol														
				0.25 hours			3 hours			6 hours			24 hours			48 hours		
Patient	SU_{IT}	SU_{REF}	R	SU_{IT}	SU_{REF}	R	SU_{IT}	SU_{REF}	R	SU_{IT}	SU_{REF}	R	SU_{IT}	SU_{REF}	R	SU_{IT}	SU_{REF}	R
1	197	118	1.7	163	112	1.5	130	86	1.5	127	80	1.6	173	94	1.8	213	119	1.8
2	171	108	1.6				167	90	1.9	133	83	1.6	123	104	1.2	155	105	1.5
3	134	112	1.2				125	91	1.4	146	94	1.5	154	125	1.2	145	119	1.2
4	195	110	1.8				169	89	1.9	183	93	2.0	194	118	1.6	185	122	1.5
5	204	100	2.0				151	85	1.8	150	75	2.0	204	111	1.8	201	106	1.9
6	198	132	1.5				143	90	1.6	135	83	1.6	179	96	1.9	207	113	1.8
7	216	109	2.0	201	111	1.8	153	93	1.6	174	86	2.0	211	114	1.9	197	106	1.9
8	178	99	1.8		64		146	64	2.3	137	79	1.7	155	93	1.7	215	109	2.0
9	142	99	1.4	131	80	1.6	148	83	1.8				131	77	1.7	134	77	1.7
10	194	96	2.0	220	106	2.1	175	77	2.3				226	98	2.3	209	107	2.0
11	192	97	2.0	195	97	2.0	205	88	2.3	177	77	2.3	194	102	1.9	205	95	2.2
12	185	109	1.7	216	112	1.9	171	105	1.6	166	95	1.8	173	101	1.7	208	110	1.9
13	150	107	1.4	180	98	1.8	139	90	1.5	170	105	1.6	158	110	1.4		114	
14	198	100	2.0	220	107	2.1	181	83	2.2	166	83	2.0	193	94	2.1	214	115	1.9
15	210	114	1.9	169	97	1.7	174	89	2.0	168	83	2.0	166	103	1.6	194	105	1.9
16	156	89	1.8	194	105	1.8	154	78	2.0	149	88	1.7	181	112	1.6	167	104	1.6
17	141	108	1.3	123	110	1.1	126	93	1.4	111	101	1.1	149	114	1.3	155	107	1.4
18	101	76	1.3				138	80	1.7	156	100	1.6	193	95	2.0	96	76	1.3
19	158	103	1.5				159	95	1.7	139	88	1.6	179	91	2.0	149	96	1.5
20	157	93	1.7	152	92	1.7	153	87	1.8	157	93	1.7	156	96	1.6	145	100	1.4
21	175	105	1.7	163	99	1.7	135	88	1.5				173	109	1.6	166	110	1.5
22	183	98	1.9	170	91	1.9	134	68	2.0	127	71	1.8	151	86	1.8	166	93	1.8
23	187	110	1.7	185	95	1.9	187	99	1.9	194	97	2.0	210	120	1.7	189	129	1.5
24	167	93	1.8							158	88	1.8	183	93	2.0	148	96	1.6

**Mean ± STD**	**174 ± 28**	**103 ± 11**	**1.7 ± 0.2**	**179 ± 30**	**98 ± 13**	**1.8 ± 0.3**	**155 ± 21**	**86 ± 9**	**1.8 ± 0.3**	**153 ± 21**	**87 ± 9**	**1.8 ± 0.3**	**175 ± 26**	**102 ± 12**	**1.7 ± 0.3**	**177 ± 32**	**105 ± 13**	**1.7 ± 0.2**

SU = signal unit; IT = inferior turbinate; REF = vitreous body of the eye; R = signal units within inferior turbinate divided by signal units within REF; STD = standard deviation.

Supplementary Table 5. Signal intensity within nasal septum and reference tissue including normalized signal unit ratios, before (Pre) and after intrathecal gadobutrol

	Pre contrast			Time after intrathecal gadobutrol														
				0.25 hours			3 hours			6 hours			24 hours			48 hours		
**Patient**	**SU$_{NS}$**	**SU$_{REF}$**	**R**	**SU$_{NS}$**	**SU$_{REF}$**	**R**	**SU$_{NS}$**	**SU$_{REF}$**	**R**	**SU$_{NS}$**	**SU$_{REF}$**	**R**	**SU$_{NS}$**	**SU$_{REF}$**	**R**	**SU$_{NS}$**	**SU$_{REF}$**	**R**
1	175	118	1.5	177	112	1.6	124	86	1.5	118	80	1.5	168	94	1.8	195	119	1.6
2	185	108	1.7				167	90	1.9	150	83	1.8	130	104	1.3	176	105	1.7
3	143	112	1.3				136	91	1.5		94		148	125	1.2	147	119	1.2
4	193	110	1.8				152	89	1.7	146	93	1.6	204	118	1.7	211	122	1.7
5	154	100	1.5				116	85	1.4	118	75	1.6	161	111	1.5	155	106	1.5
6	192	132	1.5				136	90	1.5	148	83	1.8	190	96	2.0	211	113	1.9
7	199	109	1.8	196	111	1.8	156	93	1.7	215	86	2.5	194	114	1.7	202	106	1.9
8	210	99	2.1	175	64	2.7	176	64	2.8	163	79	2.1	186	93	2.0	257	109	2.4
9	164	99	1.7	164	80	2.1	134	83	1.6				152	77	2.0	148	77	1.9
10	199	96	2.1	204	106	1.9	127	77	1.7				205	98	2.1	201	107	1.9
11	215	97	2.2	205	97	2.1	206	88	2.3	185	77	2.4	211	102	2.1	211	95	2.2
12	173	109	1.6	179	112	1.6	175	105	1.7	142	95	1.5	158	101	1.6	174	110	1.6
13	172	107	1.6	186	98	1.9	160	90	1.8	211	105	2.0	189	110	1.7		114	
14	210	100	2.1	217	107	2.0	165	83	2.0	139	83	1.7	209	94	2.2	208	115	1.8
15	166	114	1.5	144	97	1.5	149	89	1.7	153	83	1.9	154	103	1.5	156	105	1.5
16	165	89	1.9	200	105	1.9	136	78	1.7	145	88	1.7	166	112	1.5	202	104	2.0
17	154	108	1.4	121	110	1.1	112	93	1.2	112	101	1.1	165	114	1.4	121	107	1.1
18	104	76	1.4				131	80	1.6	132	100	1.3	175	95	1.9	120	76	1.6
19	138	103	1.3				139	95	1.5	134	88	1.5	113	91	1.2	128	96	1.3
20	197	93	2.1	200	92	2.2	193	87	2.2	195	93	2.1	217	96	2.3	195	100	2.0
21	167	105	1.6	173	99	1.8	140	88	1.6				178	109	1.6	162	110	1.5
22	150	98	1.5	156	91	1.7	151	68	2.2	127	71	1.8	167	86	2.0	164	93	1.8
23	192	110	1.7	201	95	2.1	182	99	1.8	203	97	2.1	217	120	1.8	176	129	1.4
24	177	93	1.9							190	88	2.2	191	93	2.1	186	96	1.9

**Mean ± STD**	**175 ± 26**	**103 ± 11**	**1.7 ± 0.3**	**181 ± 26**	**98 ± 13**	**1.9 ± 0.4**	**150 ± 24**	**86 ± 9**	**1.8 ± 0.4**	**156 ± 32**	**87 ± 9**	**1.8 ± 0.4**	**177 ± 27**	**102 ± 12**	**1.8 ± 0.3**	**178 ± 34**	**105 ± 13**	**1.7 ± 0.3**

SU = signal unit; NS = nasal septum; REF = vitreous body of the eye; R = signal units within nasal septum divided by signal units within REF; STD = standard deviation.

Supplementary Table 6. Signal intensity within grey matter of straight gyrus and reference tissue including normalized signal unit ratios, before (Pre) and after intrathecal gadobutrol

Pre contrast				Time after intrathecal gadobutrol														
				0.25 hours			3 hours			6 hours			24 hours			48 hours		
Patient	SU_{GM}	SU_{REF}	R	SU_{GM}	SU_{REF}	R	SU_{GM}	SU_{REF}	R	SU_{GM}	SU_{REF}	R	SU_{GM}	SU_{REF}	R	SU_{GM}	SU_{REF}	R
1	176	118	1.5	174	112	1.6	143	86	1.7	147	80	1.8	241	94	2.6	228	119	1.9
2	147	108	1.4				134	90	1.5	134	83	1.6	192	104	1.9	181	105	1.7
3	158	112	1.4				154	91	1.7	169	94	1.8	230	125	1.8	176	119	1.5
4	163	110	1.5				122	89	1.4	136	93	1.5	220	118	1.9	179	122	1.5
5	167	100	1.7				111	85	1.3	133	75	1.8	251	111	2.3	194	106	1.8
6	204	132	1.6				146	90	1.6	197	83	2.4	280	96	2.9	228	113	2.0
7	137	109	1.3	158	111	1.4	119	93	1.3	171	86	2.0	225	114	2.0	173	106	1.6
8	199	99	2.0		64		151	64	2.4	179	79	2.3	264	93	2.8	279	109	2.6
9	167	99	1.7	137	80	1.7	138	83	1.7				180	77	2.3	151	77	2.0
10	162	96	1.7	177	106	1.7	145	77	1.9				249	98	2.5	209	107	1.9
11	73	97	0.8	76	97	0.8	89	88	1.0	103	77	1.3	178	102	1.8	136	95	1.4
12	194	109	1.8	197	112	1.8	205	105	2.0	184	95	1.9	269	101	2.7	266	110	2.4
13	177	107	1.7	142	98	1.5	137	90	1.5	241	105	2.3	226	110	2.1		114	
14	181	100	1.8	176	107	1.7	149	83	1.8	182	83	2.2	241	94	2.6	241	115	2.1
15	172	114	1.5	141	97	1.5	148	89	1.7	161	83	2.0	238	103	2.3	198	105	1.9
16	151	89	1.7	162	105	1.5	120	78	1.5	162	88	1.9	186	112	1.7	173	104	1.7
17	149	108	1.4	143	110	1.3	174	93	1.9	226	101	2.2	239	114	2.1	190	107	1.8
18	126	76	1.7				110	80	1.4	169	100	1.7	230	95	2.4	153	76	2.0
19	170	103	1.7				189	95	2.0	215	88	2.5	245	91	2.7	277	96	2.9
20	206	93	2.2	185	92	2.0	186	87	2.1	196	93	2.1	247	96	2.6	213	100	2.1
21	146	105	1.4	142	99	1.4	135	88	1.5				206	109	1.9	166	110	1.5
22	143	98	1.5	128	91	1.4	121	68	1.8	164	71	2.3	243	86	2.8	193	93	2.1
23	146	110	1.3	144	95	1.5	163	99	1.6	199	97	2.1	193	120	1.6	158	129	1.2
24	150	93	1.6							193	88	2.2	176	93	1.9	164	96	1.7

**Mean ± STD**	**161 ± 28**	**103 ± 11**	**1.6 ± 0.3**	**152 ± 29**	**98 ± 13**	**1.5 ± 0.3**	**143 ± 28**	**86 ± 9**	**1.7 ± 0.3**	**174 ± 33**	**87 ± 9**	**2.0 ± 0.3**	**227 ± 30**	**102 ± 12**	**2.3 ± 0.4**	**197 ± 40**	**105 ± 13**	**1.9 ± 0.4**

SU = signal unit; GM = grey matter of straight gyrus; REF = vitreous body of the eye; R = signal units within grey matter of straight gyrus divided by signal units within REF; STD = standard deviation.

Supplementary Table 7. Signal intensity within deep white matter of frontal lobe and reference tissue including normalized signal unit ratios, before (Pre) and after intrathecal gadobutrol

	Pre contrast			Time after intrathecal gadobutrol														
				0.25 hours			3 hours			6 hours			24 hours			48 hours		
Patient	SU_{WM}	SU_{REF}	R	SU_{WM}	SU_{REF}	R	SU_{WM}	SU_{REF}	R	SU_{WM}	SU_{REF}	R	SU_{WM}	SU_{REF}	R	SU_{WM}	SU_{REF}	R
1	216	118	1.8	207	112	1.9	179	86	2.1	172	80	2.2	233	94	2.5	239	119	2.0
2	227	108	2.1				184	90	2.1	165	83	2.0	227	104	2.2	227	105	2.2
3	193	112	1.7				168	91	1.8	176	94	1.9	226	125	1.8	207	119	1.7
4	230	110	2.1				162	89	1.8	166	93	1.8	249	118	2.1	234	122	1.9
5	229	100	2.3				159	85	1.9	174	75	2.3	242	111	2.2	266	106	2.5
6	238	132	1.8				171	90	1.9	179	83	2.2	230	96	2.4	259	113	2.3
7	216	109	2.0	229	111	2.1	159	93	1.7	174	86	2.0	229	114	2.0	229	106	2.2
8	216	99	2.2		64		161	64	2.5	177	79	2.2	207	93	2.2	261	109	2.4
9	200	99	2.0	180	80	2.3	174	83	2.1				202	77	2.6	206	77	2.7
10	207	96	2.2	240	106	2.3	191	77	2.5				253	98	2.6	261	107	2.4
11	177	97	1.8	197	97	2.0	182	88	2.1	176	77	2.3	204	102	2.0	208	95	2.2
12	230	109	2.1	221	112	2.0	243	105	2.3	217	95	2.3	227	101	2.2	258	110	2.4
13	250	107	2.3	212	98	2.2	192	90	2.1	215	105	2.1	217	110	2.0		114	
14	240	100	2.4	218	107	2.0	193	83	2.3	178	83	2.1	242	94	2.6	284	115	2.5
15	226	114	2.0	190	97	2.0	184	89	2.1	177	83	2.1	230	103	2.2	246	105	2.4
16	193	89	2.2	210	105	2.0	165	78	2.1	189	88	2.2	212	112	1.9	230	104	2.2
17	215	108	2.0	178	110	1.6	171	93	1.8	171	101	1.7	224	114	2.0	245	107	2.3
18	178	76	2.3				169	80	2.1	184	100	1.8	214	95	2.3	194	76	2.6
19	230	103	2.2				224	95	2.4	213	88	2.4	235	91	2.6	293	96	3.0
20	209	93	2.2	181	92	2.0	179	87	2.1	170	93	1.8	197	96	2.1	219	100	2.2
21	203	105	1.9	184	99	1.9	160	88	1.8				210	109	1.9	234	110	2.1
22	192	98	2.0	180	91	2.0	152	68	2.2	160	71	2.3	209	86	2.4	227	93	2.5
23	173	110	1.6	157	95	1.6	179	99	1.8	177	97	1.8	207	120	1.7	208	129	1.6
24	179	93	1.9							163	88	1.8	158	93	1.7	179	96	1.9

**Mean ± STD**	**211 ± 22**	**103 ± 11**	**2.1 ± 0.2**	**199 ± 23**	**98 ± 13**	**2.0 ± 0.2**	**178 ± 21**	**86 ± 9**	**2.1 ± 0.2**	**179 ± 16**	**87 ± 9**	**2.1 ± 0.2**	**220 ± 20**	**102 ± 12**	**2.2 ± 0.3**	**235 ± 28**	**105 ± 13**	**2.3 ± 0.3**

SU = signal unit; WM = deep white matter of frontal lobe; REF = vitreous body of the eye; R = signal units within deep white matter of frontal lobe divided by signal units within REF; STD = standard deviation.
